# Cost-effectiveness of tyrosine kinase inhibitor treatment strategies for chronic myeloid leukemia in South Africa

**DOI:** 10.3389/fphar.2024.1511603

**Published:** 2025-01-07

**Authors:** Rochelle Woudberg, Edina Sinanovic

**Affiliations:** Health Economics Unit, School of Public Health, University of Cape Town, Cape Town, South Africa

**Keywords:** chronic myeloid leukemia, tyrosine kinase inhibitors, economic evaluation, cost-effectiveness, South Africa

## Abstract

**Background:**

The treatment of chronic myeloid leukemia through tyrosine kinase inhibitors (TKIs) has achieved promising efficacy and safety outcomes, however the costs are associated with a substantial economic burden. The objective of this study was to develop a Markov model with a 20-year time horizon to assess the cost effectiveness of TKIs from a public healthcare system perspective in South Africa.

**Methods:**

We constructed a Markov model to compare three strategies in which treatment was initiated with either imatinib, nilotinib, or dasatinib. Treatment was switched to another TKI in the case of intolerance or resistance to the initial TKI. Effectiveness and utility data were obtained from published literature. Cost data was obtained from local sources for generic imatinib and branded second-generation TKIs and based on national tariffs. Outcomes were reported in total costs and quality-adjusted life years (QALYs). Outcomes were based on calculated incremental cost effectiveness ratios (ICERs) and compared to a willingness-to-pay (WTP) threshold. Sensitivity analyses were conducted to determine the robustness of the model outcomes.

**Results:**

The base-case results showed that imatinib was favored over nilotinib and dasatinib by having the lowest cost at $120 719.55 and providing 5.93 QALYs. Compared to imatinib strategy, nilotinib had an ICER of $26 620.27 per QALY and dasatinib had an ICER of $35 934.94 per QALY, both exceeding the WTP threshold of $18 760 per QALY gained. The sensitivity analysis indicated the robustness of the results.

**Conclusion:**

Imatinib remains the most cost-effective first-line treatment for adults diagnosed with CML in South Africa, with a high probability of being cost-effective across a range of WTP thresholds. Nilotinib and Dasatinib, though offering clinical benefits, their affordability remains a challenge within the current healthcare system and should remain reserved for second-line treatment.

## 1 Introduction

Chronic myeloid leukemia (CML) is a clonal myeloproliferative disorder of hematopoietic stem cells and is considered the third most common type of leukemia (Flis and Chojnacki, 2019). CML is characterized by a translocation between chromosome 9 and 22 that gives rise to the Philadelphia chromosome (Ph), causing the development of the *BCR-ABL1* oncogene that results in the constitutive tyrosine kinase activity that leads to dysregulated cell proliferation and apoptosis (Hochhaus et al., 2011). In 2021, an estimated prevalence of 0.125 per 10,000 individuals was reported and an incidence of 1–1.5 per 100,000 individuals, accounting for approximately 15% of adult leukemia cases worldwide (Agrawal et al., 2022; Ning et al., 2020).

The natural history of CML consists of three progressive phases based on the number of immature white blood cells in the blood or bone marrow: a chronic phase (CP), an accelerated phase (AP) and a blast phase (BP). The median age at diagnosis of CML is approximately 57–60 years and is more common among males (Ning et al., 2020; Hoglund et al., 2015). However, in South Africa, CML patients tend to be diagnosed at an earlier age, with a mean age of 42.5 years, which is consistent with the mean age of 38.5 years reported in other low- and middle-income countries (LMICs) (Mendizabal et al., 2013). The majority (90%–95%) of CML patients are diagnosed in the CP, with up to 40% being asymptomatic (Deininger et al., 2020). In South Africa, it has been reported that 83.8% of CML patients are diagnosed in CP, while 16.2% are diagnosed in the more advanced AP or BP (Sikhipha et al., 2020). The median survival for patients with untreated CML is 4–5 years. If untreated, a patient will progress from CP to AP within 3–5 years, while often remaining to be asymptomatic (Deininger et al., 2020; Thompson et al., 2015). The transition to AP lasts about 3–9 months and is experienced by approximately 60%–80% of patients (Thompson et al., 2015; Silver, 2009). The final transitions from AP to BP occurs within 4–6 months and the median survival being only 3–6 months (Silver, 2009). Moreover, in South Africa, the overall survival for patients in AP/BP has been reported to be just 7 months, with the main causes of mortality being sepsis and relapsed refractory disease. Despite these challenges, a study of CML patient outcomes in South Africa found that two-thirds of patients achieved an optimal response at 18 months, highlighting the potential benefits of targeted therapies (Sikhipha et al., 2020). Although each phase has distinct differences in both the clinical and pathological definitions, the treatment options for CML depend on the phase of the disease, other prognostic factors, and the availability of a stem cell donor.

Tyrosine kinase inhibitors (TKIs) have become the treatment of choice across different therapy lines for CML. Since the integration of TKIs into the treatment pathway, the annual mortality of CML has decreased from 10%–20% to 1%–2% globally, transforming CML from a fatal cancer into a manageable disease with a significantly improved life expectancy (Jabbour and Kantarjian, 2016; Hirt et al., 2019). In 2001, imatinib mesylate was approved as the TKI for CML treatment with Phase II trial results showing relatively high hematological and cytogenetic response rates (Kantarjian et al., 2002; Sawyers et al., 2002). This was rapidly followed by second-generation drugs including nilotinib and dasatinib in 2007, and more recently bosutinib and the third-generation drug ponatinib in 2012. Currently, the FDA has approved the imatinib, nilotinib, dasatinib, and most recently bosutinib as the first-line treatment for patients with CML in the chronic phase (CML-CP) (Radich et al., 2018), however in South Africa imatinib is approved as the first-line treatment, nilotinib and dasatinib are listed as second-line treatments and bosutinib remains unavailable.

Resistance, tolerability issues or lack of response to TKI treatments requires a switch in TKI treatment to limit the risk of disease progression which causes both clinical and economic challenges to CML management. It has been estimated that over 25% of CML patients switch TKIs at least once due to resistance or intolerance (Patel et al., 2017). Furthermore, while switching between TKI lines the rate of treatment failure increases (Zhang et al., 2020). South Africa’s unique epidemiological profile, including a younger patient population and reported comorbidities in CML patients such as hypertension (67.6%), HIV (10.8%), and diabetes mellitus (8.1%), further complicates disease management (Sikhipha et al., 2020). Additionally, in South Africa, systematic issues such as treatment interruptions, limited adherence due to high out-of-pocket costs, and long travel distances to tertiary centers exacerbate the risk of disease progression (McGarry et al., 2016). These challenges underscore the need for cost-effective and accessible treatment options tailored to the local context.

Economic evaluations provide a solution for selecting clinical interventions in healthcare and assist decision makers appraise the comparative effectiveness and projected costs associated with different interventions. The cost-effectiveness of CML treatment has been of interest in many countries and several cost-effectiveness analyses have been conducted, however, these have been limited to Middle- and High-Income countries (Marsh et al., 2014; Pavey et al., 2012; Rochau et al., 2014; Fu et al., 2018). To our knowledge, no previous cost-effectiveness analysis for the treatment of CML in South Africa has been evaluated. To address this scarcity of health-economic data in LMICs and compare approved TKIs, we developed a Markov model to estimate the 20-year cost effectiveness of three TKIs (imatinib, nilotinib, and dasatinib) as first-line therapy treatment strategies for patients with CML in South Africa.

## 2 Methods

### 2.1 Study design

A cost-effectiveness analysis was performed using a Markov cohort model from a South African public healthcare provider perspective (Briggs and Sculpher, 1998). A hypothetical cohort of 1,000 newly diagnosed adult patients with CML who would be starting therapy on a first line TKI. In this cohort patients could be assigned to three different initial treatment strategies: 1) imatinib, 2) nilotinib, or 3) dasatinib as first-line therapy.

The cohorts began in the CML-CP first-line TKI treatment state and progressed to other health states according to transition probabilities derived from published literature. The cycle length was 12 months, and a 20-year time frame was chosen. This cycle length was chosen as a balance between keeping the model as simple as possible to allow implementation, while being sufficiently finely grained to reflect the clinical course and management of CML. Half-cycle corrections and discounting (5% to costs and effects, as per the SA HTA guidelines) were applied (Wilkinson et al., 2022). The health outcomes for this study were total costs and quality-adjusted life years (QALYs). We varied probabilities, cost estimates, and QALYs to account for uncertainty in our model parameters. The model development and all analyses were conducted using Microsoft^©^ Excel (Microsoft Corporation, Redmond, WA, USA). Ethical approval was obtained by the University of Cape Town (UCT) Human Research Ethics Committee (ref. no. 923/2023).

### 2.2 Markov model

**FIGURE 1:**
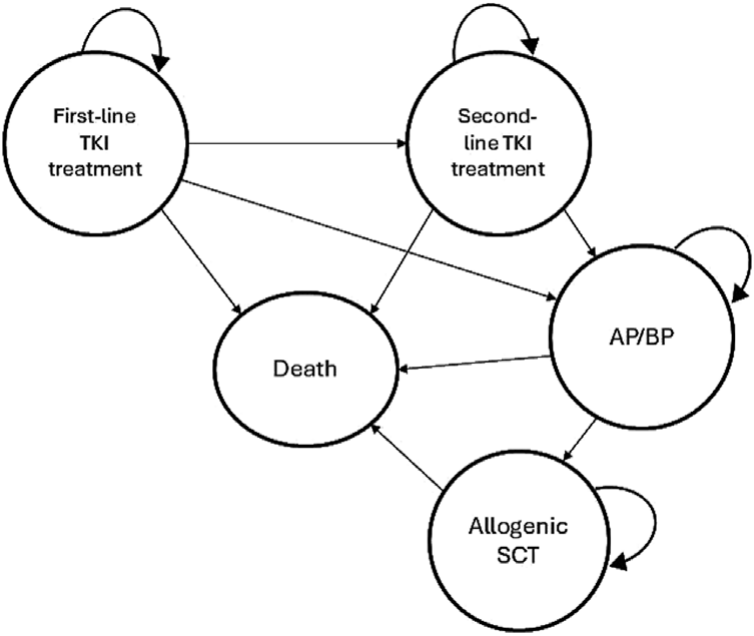
Markov model structure of the cost-effectiveness analysis.

The Markov model was designed based on the clinical setting and the recommended Clinical Practice Guidelines in South Africa (Louw et al., 2011). This model structure simulates a cohort of patients with CML-CP transitioning through the various treatment pathways and consists of 5 mutually exclusive health states: First-line TKI treatment (state 1), second-line TKI treatment (state 2), progression to AP/BP (state 3), receiving an allogeneic stem cell transplantation (SCT) (state 4), and death (state 5). The Markov model structure used for all three strategies is shown in Figure 1.

In this model, it was assumed that all patients start with a first-line TKI treatment in CML-CP. Patients can then either remain in the same health state or switch to a second-line TKI treatment in CML-CP after first-line TKI failure or intolerance. Subsequently, patients can remain in the second-line TKI treatment or progress to AP/BP. Treatment failure leading to a TKI switch was defined as less than complete cytogenic response (CCyR) at 12 months or intolerance of the TKI due to adverse events (AEs). In the imatinib strategy, after failure the TKI was switched to either of the second-generation TKIs (nilotinib or dasatinib) in equal proportions (Padula et al., 2016). After the failure of either nilotinib or dasatinib, it was assumed that a switch to imatinib was made in 15% of patients due to intolerance and a switch to the other second-generation TKIs were made in 85% of patients due to resistance (Padula et al., 2016; Yamamoto et al., 2019). In the AP/BP state patients can either remain on the current treatment or undergo an allogeneic SCT. Transition to death is possible from any health state due to all-cause mortality, while dying from CML is only possible in BP (Padula et al., 2016). It was assumed that all patients would be in only one health state in any 12-month single cycle.

### 2.3 Model input parameters

#### 2.3.1 Transition probabilities

The annual transition probabilities among the Markov health states were estimated based on data from the results of clinical trials. Transition probabilities were derived from survival rates and causes of death for newly diagnosed CML patients comparing imatinib, nilotinib, and dasatinib.

We used the survival data from ENESTnd and DASISION trials to capture overall survival of the nilotinib and dasatinib strategies, respectively (Hochhaus et al., 2016; Kantarjian et al., 2012). For the imatinib strategy, we used survival data from the imatinib treatment arm of the ENESTnd trial (Kantarjian et al., 2012). The efficacy and rates for therapy switches are based on 12-month CCyR, which is defined as “absence of the Ph^+^ chromosome among at least 20 cells in metaphase in the bone marrow” (Alattar et al., 2011). Overall survival rates for allogeneic SCT were not available for South Africa, as SCT is not a treatment option for many patients, especially in limited resource settings. The probability that patients would proceed to allogeneic SCT after progression to AP/BP was therefore calculated based on the results of the IRIS study (Hochhaus et al., 2017). All rates used in the study were converted to annual probabilities. The transition probabilities defined in the model and their plausible ranges and distributions for sensitivity analysis are shown in Table 1.

**TABLE 1:** Transition probabilities of health states.

Probabilities	Values or formulae		Reference
	Baseline	Range for sensitivity analysis	
*CCyR*			
Imatinib	0.67	0.60–0.81	Hochhaus et al. (2016), Larson et al. (2012)
Nilotinib	0.76	0.63–0.85	Hochhaus et al. (2016), Larson et al. (2012)
Dasatinib	0.80	0.68–0.92	Kantarjian et al. (2012), Kantarjian et al. (2010)
*TKI Switch rates*			
Imatinib to nilotinib	0.50		Padula et al. (2016)
Imatinib to dasatinib	0.50		Padula et al. (2016)
Nilotinib to imatinib	0.15		Padula et al. (2016)
Nilotinib to dasatinib	0.85		Padula et al. (2016)
Dasatinib to imatinib	0.15		Padula et al. (2016)
Dasatinib to nilotinib	0.85		Padula et al. (2016)
*AP/BP*			
Imatinib	0.048	0.42–0.54	Hochhaus et al. (2017)
Nilotinib	0.019	0.017–0.021	Hochhaus et al. (2016)
Dasatinib	0.043	0.037–0.049	Kantarjian et al. (2012), Cortes et al. (2016)
*Overall survival*			
Imatinib	0.977	0.83–1	Hochhaus et al. (2017)
Nilotinib	0.933	0.79–1	Hochhaus et al. (2016), Larson et al. (2012)
Dasatinib	0.931	0.81–1	Cortes et al. (2016)
AP/BP	0.052	0.046–0.060	Hochhaus et al. (2017), Larson et al. (2012), Cortes et al. (2016)
*Allogeneic SCT*			
Receiving a SCT	0.038	0–1	Hochhaus et al. (2017)
Survival post-SCT	0.429	0.354–0.504	Hochhaus et al. (2017)
Death post-SCT*	0.571	0.496–0.646	Estimated

*Indicates remainder probability, adding up to 1.0 for all subnodes. β distributions were used for probabilistic sensitivity analysis.

Abbreviations: *AP/BP*, accelerated phase/blast phase, *CCyR* complete cytogenetic response, *SCT*, stem cell transplant; *TKI*, tyrosine kinase inhibitor.

#### 2.3.2 Costs

The analysis was performed from the public healthcare payer’s perspective and therefore included only direct medical costs. The estimated costs for each strategy were comprised of TKI drug costs, consultation and hospitalisation costs, laboratory tests, and per-event costs for AEs and allogeneic SCT procedures (Table 2). Patients were assumed to receive standard doses of imatinib (400 mg once daily), nilotinib (400 mg twice daily) and dasatinib (100 mg once daily). The costs for TKIs, consultations, hospitalisations, laboratory tests, and allogeneic SCT were defined according to standard practice in South Africa (Louw et al., 2011).

**TABLE 2:** Costs used for the analysis of cost-effectiveness.

	Baseline value (US$)	Range for sensitivity analysis	Reference
*Drug costs – per year*			
Imatinib (Imatinib accord)	4,634.40	2,317.20–6,951.60	Health (2024)
Nilotinib (Tasigna)	24,517.94	12,258.97–36,776.91	Health (2024)
Dasatinib (Sprycel)	10,790.80	5,395.40–16,186.20	Health (2024)
*Monitoring and follow-up – per year*			
Consultation/Hospitalization	94.91	47.46–142.37	Africa (2024)
Monitoring and laboratory tests	806.25	403.12–1,209.38	NHLS (2018)
After allogeneic SCT, first year	543.55	271.78–815.32	Health (2024); Africa (2024)
After allogeneic SCT, subsequent years	427.80	213.90–641.70	Health (2024); Africa (2024)
*Per-event costs*			
Allogeneic SCT, initial cost	14,722.43	7,361.21–22,083.64	Africa (2024); NHLS (2018); SANBS (2024)
*Adverse events*			
Non-haematological costs	23.53	11.75–35.29	Health (2024)
Haematological costs			
Neutropenia	358.50	179.25–537.75	Health (2024); Africa (2024)
Thrombocytopenia	1,164.28	582.14–1,746.42	Africa (2024); SANBS (2024)
Anaemia	799.39	399.70–1,199.08	Africa (2024); SANBS (2024)

γ distributions were used in the probabilistic sensitivity analysis. Using official 2024 exchange rates, US$1 = ZAR, 17.85.

Abbreviations: *SCT*, stem cell transplantation; *QALY*, quality-adjusted life-year; *y,* year.

The drug costs were obtained from the database of medicine prices (Health, 2024). The base line costs of TKIs were determined based on the current branded TKIs for nilotinib and dasatinib, and the prices of the currently available generic compounds of imatinib were used as they are prescribed for use in the public healthcare sector in South Africa. The consultation and hospitalisation costs were obtained from the SA uniform patient fee schedule (UPFS) (Africa). The costs of laboratory tests were extracted from National Health Laboratory Services (NHLS) of South Africa price lists and inflated to 2024 prices (NHLS). Additionally, for simplification, only adverse events that occurred with a frequency of at least 5% and only grades 3/4 events were considered. All costs were expressed in 2024 currency and costs in local currencies were converted to US$ using official 2024 exchange rates (US$1 = ZAR17.85).

#### 2.3.3 Utilities

Due to the absence of South Africa-specific studies on health utilities due to CML, health utilities for each health state was extracted from a published multinational CML health-state study and compared them to utilities from the IRIS trial (Hochhaus et al., 2017; Szabo et al., 2010). Utility values can range from 1 representing full health to 0 representing death (Drummond et al., 2015). All utility values were assumed to last one Markov cycle (i.e., 12 months). The utility values were used to calculate QALY endpoints, which were discounted at 5%. Table 3 shows the utilities associated with the various health states modelled.

**TABLE 3:** The utility values for the analysis of cost-effectiveness.

Health states	QALY	
	Utility value	Range
Chronic phase 1st line	0.89	(0.78–0.94)
Chronic phase 2nd line	0.75	(0.57–0.85)
AP/BP	0.22	(0.07–0.34)
Allogeneic SCT, within 1 year	0.60	(0.51–0.69)
Allogeneic SCT, after 1 year	0.85	(0.723–0.978)
Death	0	0

QALYs, were extracted from Szabo et al. (2010). β distributions were used for utilities in probabilistic sensitivity analysis. In general, QALYs, range from 0.0 to 1.0, where 0.0 represents death and 1.0 represents full health over 1 year period.

Abbreviations: *AP/BP*, accelerated phase/blast phase; *SCT*, stem cell transplantation; *QALY*, quality-adjusted life-year; *y*, year.

### 2.4 Cost-effectiveness analysis

In the base-case analysis, total costs and total QALYs for each strategy were calculated to estimate the incremental cost-effectiveness of nilotinib and dasatinib compared to imatinib. The incremental cost-effectiveness ratio (ICER) was calculated as the incremental cost per QALY gained between the groups compared.

The one-to-three times GDP *per capita* thresholds recommendation from the World Health Organization (WHO) has been the most widely used threshold for determining cost effectiveness of interventions, particularly in LMICs (Leech et al., 2018). The willingness-to-pay (WTP) threshold value for QALY was set as three times South Africa’s GDP *per capita* in 2023 (GDP *per capita* = $6,253.20, WTP = $18 760 per QALY gained). The ICERs for nilotinib and dasatinib were compared against this WTP threshold to determine whether these therapies offer good value for money relative to imatinib. Interventions with ICERs below this threshold were considered cost-effective.

### 2.5 Sensitivity analysis

Deterministic and probabilistic sensitivity analyses were conducted for all variables to assess the robustness of the results. In the deterministic one-way sensitivity analysis, parameters were varied and their effects on the ICER were observed. All parameters were assigned with lower and upper limits varied in their 95% confidence intervals or ±50% of the base case value when the 95% confidence interval was not available as per the SA HTA guideline (Wilkinson et al., 2022). The rate of patients switching to second-line TKI treatment for imatinib strategy was varied from the 50%/50% assumption for nilotinib/dasatinib up to 100% for each drug. However, for transition probabilities, we capped the highest probability at 1 regardless of the results from the +50% variation and drug prices were varied according to literature. The discount rates were varied between 0% and 10% for the analysis as per the HTA methods guide (Wilkinson et al., 2022).

In addition, a probabilistic sensitivity analysis (PSA) was performed through a 1,000 Monte Carlo simulation, with gamma distributions applied to costs and beta distributions applied to probabilities and utilities. The results from the 1,000 iterations were scattered to form a cost-effectiveness scatter plot. A cost-effectiveness acceptability curve (CEAC) was plotted to determine the probability that a given treatment would be cost-effective at different WTP ($/QALY) thresholds. The PSA was used to randomly select parameter values from their assumed distributions in order to provide more realistic CML population result.

## 3 Results

### 3.1 Base-case analysis

The cost-effectiveness of the three strategies of TKI treatment for CML over the 20-years is shown in Table 4. The base case results showed that the total cost of patients treated with TKI therapy using imatinib, nilotinib, and dasatinib was $120 719.55, $169 861.41, and $180 774.97, respectively. The total costs were estimated to be increased by $49 141.86 in nilotinib compared to imatinib and $60 055.42 increase in dasatinib compared to imatinib. The total QALYs associated with treatment were 5.93, 7.78, and 7.60 respectively, implying the effectiveness was 1.85 QALY improved in nilotinib compared to imatinib and a 1.67 QALY improved in dasatinib compared to imatinib. The estimated ICER for nilotinib versus imatinib was $26 620.27 per QALY gained while that for dasatinib versus imatinib was $35 934.94 per QALY gained. Neither of the two strategies met a WTP threshold ($18 760 per QALY) based on the ICER.

**TABLE 4:** Base case cost-effectiveness analysis results.

Strategy	Total		Incremental		ICER
	Costs (US$)	QALYs	Costs (US$)	QALYs	
Imatinib	120,719.55	5.93	-	-	-
Nilotinib	169,861.41	7.78	49,141.86	1.85	26,620.27
Dasatinib	180,774.97	7.60	60,055.42	1.67	35,934.94

ICER, incremental cost-effectiveness ratio; QALY, quality-adjusted life year.

### 3.2 Sensitivity analysis

**FIGURE 2:**
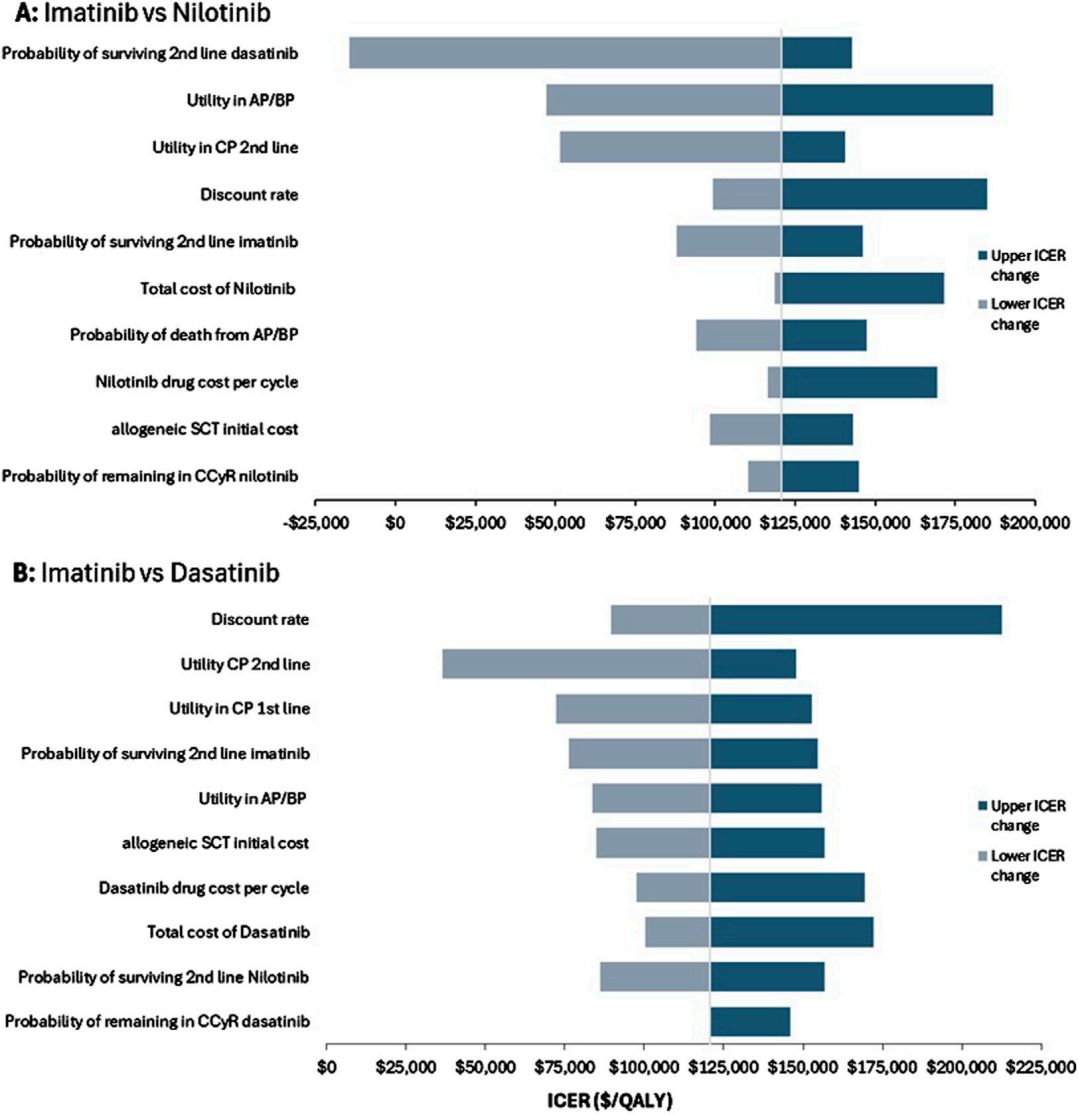
One-way deterministic sensitivity analysis. Tornado diagram for **(A)** imatinib versus nilotinib and **(B)** imatinib versus dasatinib.

In the deterministic one-way sensitivity analysis, the most influential model parameter for the comparison of imatinib versus nilotinib was the probability of surviving second-line dasatinib, followed by the utility value of patients in AP/BP. The most influential model parameter for imatinib versus dasatinib was the discount rate, followed by the utility value of patient in CP second line therapy. The tornado diagram in Figure 2 shows the changes in ICERs generated by all the sensitivity analyses parameters conducted and the robustness of the results.

**FIGURE 3:**
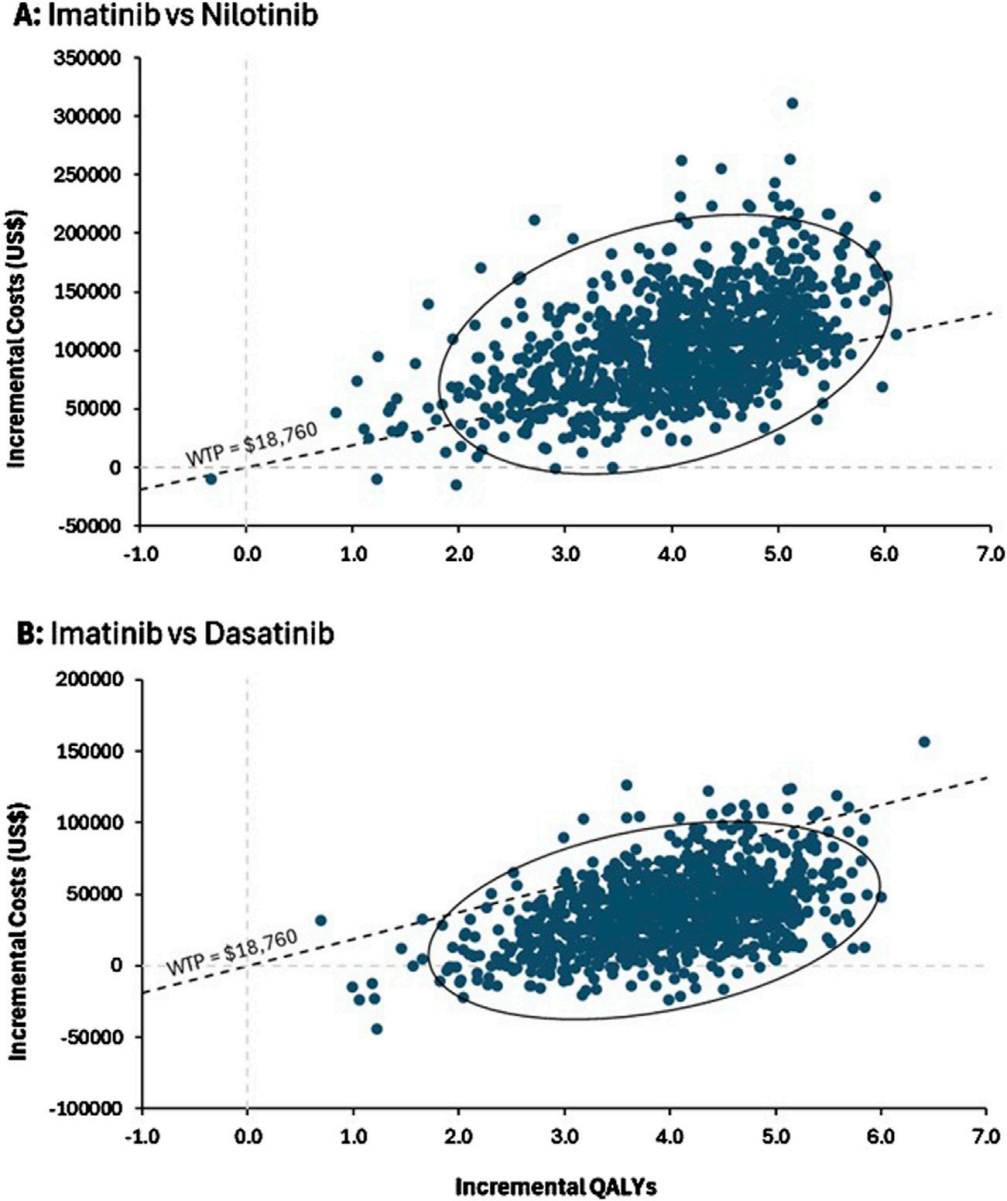
Probabilistic sensitivity analysis. ICER scatter plots for **(A)** imatinib versus nilotinib and **(B)** imatinib versus dasatinib. The confidence ellipse demonstrates the 95%CI of ICER among the simulations and the dashed diagonal line indicating the willingness-to-pay (WTP) threshold which had a slope of $18,760/QALY.

The probabilistic sensitivity analysis demonstrated that imatinib remained the most cost-effective strategy, with the lowest incremental cost compared to nilotinib and dasatinib. According to the scatter plots, dasatinib showed a 94% probability of being cost-effective under the set WTP threshold, while nilotinib had only a 23% probability (Figure 3).

**FIGURE 4:**
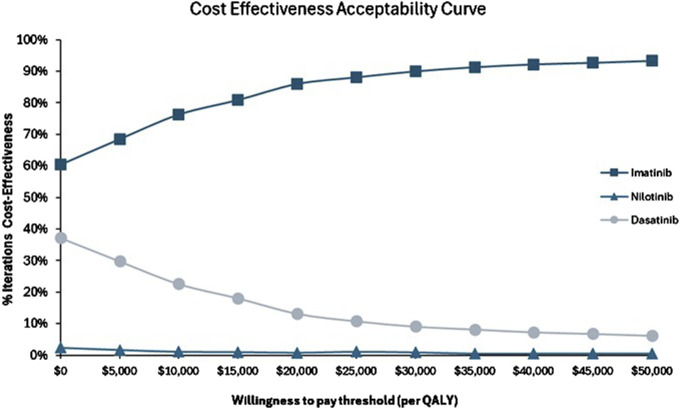
Cost-effectives acceptability curve for imatinib versus nilotinib and imatinib versus dasatinib.

The cost-effectiveness acceptability curve further indicated that imatinib had an 84% probability of being cost-effective at the WTP threshold, compared to 1% for nilotinib and 15% for dasatinib (Figure 4). These results suggest that while imatinib is generally the most favorable treatment, dasatinib may also provide value for money in a substantial number of scenarios, while nilotinib is less likely to be cost-effective overall.

## 4 Discussion

Gaining an understanding of the value of a new or alternative clinical intervention is crucial to guide rational clinical use of drugs, specifically in a limited resource setting. In this study, we performed a 20-year simulation using a Markov model to compare the cost-effectiveness of three strategies for CML patients based on initial TKI treatment. Our findings identified imatinib as the dominant strategy in terms of incremental costs per additional QALY gained. Both the nilotinib and dasatinib strategies cost more and gained more QALYs than the imatinib strategy. The lower costs associated with the imatinib results reflect the impact of price reductions due to its generic availability, positioning the first-generation TKI as a more cost-saving option at lower WTP thresholds than branded TKIs (Gorkin and Kantarjian, 2016). The incremental analysis showed that the ICERs nilotinib versus imatinib was $26 620.27 per QALY gained while that for dasatinib versus imatinib was $35 934.94 per QALY gained which exceeded the WTP threshold. Based on the base case values and potential cost-effectiveness threshold, neither nilotinib nor dasatinib, although clinically effective, are cost-effective in this setting when used as first-line therapy for adult CML patients in South Africa. The affordability of these treatments remains a significant concern for the South African healthcare system, which operates under budgetary constraints. South Africa’s total health expenditure is approximately 8.5% of GDP, with oncology representing a small fraction of this budget. While imatinib, due to its generic availability, emerges as the most cost-effective option for first-line therapy, the high costs of nilotinib and dasatinib place substantial pressure on resource allocation. The inclusion of nilotinib with an annual cost of ($24 517.92 per patient) and dasatinib ($10 790.76 per patient) would place significant pressure on limited resources, potentially diverting funding from other priority health programs such as HIV and tuberculosis. Policymakers must consider the financial burden of incorporating these TKIs into routine care for CML, especially in light of the availability of the more cost-effective generic imatinib.

While this study highlights the cost-effectiveness of TKIs, disparities in access remain a significant challenge in South Africa. Access to second-line treatments TKIs and alloSCT is limited in South Africa, particularly in the public sector, where these advanced therapies are not uniformly available (Sikhipha et al., 2020). Geographic access to healthcare facilities and lack of well-trained primary care providers are of the greatest barriers to leukemia care in South Africa, especially for rural and underserved populations. Furthermore, systemic challenges such as treatment interruptions, limited follow-up, and poor compliance may affect clinical outcomes and effectiveness of TKIs. A South African study reported poor compliance in 18.9% of patients and missed clinic appointments in 16.2%, largely due to dependence on public transport, long travel distances (up to 950 km), poor socioeconomic circumstances, and language barriers during consultations (Sikhipha et al., 2020). These factors hinder patients’ understanding of their disease and the importance of adherence. Poor adherence to imatinib therapy, for instance, can result in suboptimal treatment responses, resistance, and disease progression. Studies have shown that patients taking less than 75% of prescribed TKI doses are significantly less likely to achieve optimal cytogenetic responses (Jabbour et al., 2012). Addressing these challenges through decentralized molecular monitoring, improved patient education, and subsidized access to TKIs is critical to maximizing treatment benefits across diverse populations and geographical regions.

The probabilistic sensitivity analysis and scatter plots provided further insights into the robustness of these results. At a WTP threshold of $18 760, imatinib consistently emerges as the most cost-effective strategy, with a high probability of being cost-effective across a wide range of scenarios. These findings are aligned with current South African clinical recommended guidelines, which recommend imatinib as the first-line therapy, with nilotinib and dasatinib being reserved for second-line use in cases of intolerance or resistance to Imatinib (Louw et al., 2011). The PSA results demonstrated that a significant reduction in the cost of nilotinib or dasatinib would be required to bring them within the cost-effectiveness threshold. This highlights the importance of price negotiations or alternative funding mechanisms to make these treatments more accessible, particularly for patient subgroups that may benefit most from second-generation TKIs.

Moreover, despite dasatinib average ICER exceeding the WTP threshold, approximately 95% of the simulations for dasatinib fell below the $18,760 threshold, indicating a significant chance that dasatinib could be considered cost-effective in certain scenarios. This high percentage suggests that, despite its higher base-case ICER, dasatinib can be a cost-effective option in most scenarios and is better suited as second-line treatment due to favorable incremental QALYs provided that the drug price is decreased or if the WTP threshold was higher. This is evidence from a review study by Fu et al., in the case of imatinib treatment in CML patients who are resistant or intolerant, dasatinib is likely to be a more cost-effective strategy in middle-income countries (Fu et al., 2018). This finding highlights the potential variability in outcomes and underscores the importance of considering uncertainty in economic evaluations.

Several prior economic evaluation studies have been conducted to estimate the cost-effectiveness of TKIs for the treatment of CML (Fu et al., 2018). The study findings were consistent with findings of Padula et al., Li et al., and Rochau et al. reported that imatinib was relatively more cost effective compared to nilotinib and dasatinib (Padula et al., 2016; Li et al., 2018; Rochau et al., 2015). These studies were conducted from US, Chinese and Austrian perspectives, respectively. Moreover, in a recent decision analytic study, Shih et al. showed that generic imatinib was overwhelmingly cost-effective for attaining treatment-free remission compared to second-generation TKIs (Shih et al., 2019). Particularly, Yamamoto et al. similarly concluded that although the probability of treatment-free remission was higher for second-generation TKIs, imatinib was still more cost-effective even with the incorporation of treatment discontinuation (Yamamoto et al., 2019).

Additionally, studies have illustrated that imatinib is a cost-effective first-line treatment for newly diagnosed CP-CML patients compared to the more traditional standard of care IFN-α. The study by Reed et al. conducted in the United States demonstrated the cost-effectiveness of imatinib compared to IFN-α (Reed et al., 2008). Wolters et al. conducted a cost-effectiveness modeling study in the Netherlands, which reached similar conclusions and found that the cost-effectiveness of imatinib improves over time (Wolters et al., 2019). These findings further support the use of imatinib over interferon, which was previously considered a standard-of-care treatment before the introduction of TKIs.

Notably, all these studies were conducted in middle and/or high-income countries where cost-effectiveness thresholds are higher than in South Africa. Although the studies had similar outcomes, the outcome values differed considerably. These variations can be attributed to different methodologies approaches, varying survival assumptions, and differing WTP thresholds. Moreover, the country-specific nature of these cost-effectiveness analyses, including the unique cost structures and economic conditions of each country, restricts the generalizability of the results. Therefore, it is essential to conduct economic evaluations of specific diseases in specific countries, ensuring relevance to local pricing and healthcare needs.

The study has several limitations. First, all cost parameters were derived from the South African context specifically, which may be different from other countries. Second, data regarding utilities were unavailable specifically for the South African setting and specific to each treatment line, which is a limitation when comparing QALYs and can only be solved by conducting utility studies. This may introduce bias and add a level of uncertainty to the results. However, the robustness of our findings was confirmed through a series of sensitivity analyses, minimizing this concern. This gap underscores the need for local utility studies to better capture the quality-of-life impacts of CML treatments in this context. Third, the effectiveness data were obtained from three different clinical trials which required an indirect comparison of the drugs for each strategy. However, we made similar assumptions as were made in other CEA studies. Moreover, the cost of AEs of the three TKI drugs in this study was not calculated separately and therefore included in the total cost of treatment. The ratio of the cost of AEs to the overall cost was minor and did not affect the conclusion. The sensitivity analysis was therefore carried out to verify the stability and reliability of the model calculation. Finally, the study did not consider the impact of co-morbidities on the treatment pathway for each treatment strategy, the exclusion of this consideration was due to a lack of available data. Future research should incorporate real-world data to capture these nuances more accurately and enhance the model’s external validity.

## 5 Conclusion

The imatinib strategy was found to be both cost-saving and cost-effective as first-line TKI treatment for adult CML patients in South Africa. Second-line TKIs, particularly dasatinib, should be reserved for cases of resistance or intolerance, as current evidence suggests they may be more cost-effective in middle-income countries. However, future changes in drug pricing or healthcare policy could warrant a re-evaluation of these findings. Further research into cost-reduction strategies and patient selection criteria may be necessary to optimize the use of second-generation TKIs in South Africa.

## Data Availability

The original contributions presented in the study are included in the article/supplementary material, further inquiries can be directed to the corresponding author.

## Abbreviations

AEs, Adverse events; AP, Accelerated phase; BP, Blast phase; CCyR, Complete cytogenic response; CEAC, Cost-effectiveness acceptability curve; CML, Chronic myeloid leukemia; CML-CP, Chronic myeloid leukemia–Chronic Phase; CP, Chronic phase; ICER, Incremental cost-effectiveness ratio; HTA, Health Technology Assessment; NHLS, National Health Laboratory Services; Ph, Philadelphia chromosome; PSA, Probabilistic sensitivity analysis; QALYs, quality-adjusted life years; SA, South Africa; SCT, Stem cell transplantation; TKIs, Tyrosine kinase inhibitors; US, United States; WHO, World Health Organization; WTP, Willingness-to-pay.
